# A SIRPαFc Fusion Protein Conjugated With the Collagen-Binding Domain for Targeted Immunotherapy of Non-Small Cell Lung Cancer

**DOI:** 10.3389/fimmu.2022.845217

**Published:** 2022-03-29

**Authors:** Jiayang Liu, Zhefeng Meng, Tongyang Xu, Kudelaidi Kuerban, Songna Wang, Xuyao Zhang, Jiajun Fan, Dianwen Ju, Wenzhi Tian, Xuan Huang, Xiting Huang, Danjie Pan, Huaning Chen, Weili Zhao, Li Ye

**Affiliations:** ^1^ Minhang Hospital & Department of Biological Medicines at School of Pharmacy, Fudan University, Shanghai, China; ^2^ Shanghai Engineering Research Center of Immunotherapeutics, School of Pharmacy, Fudan University, Shanghai, China; ^3^ ImmuneOnco Biopharma (Shanghai) Co., Ltd., Shanghai, China; ^4^ Department of Medicinal Chemistry, School of Pharmacy, Fudan University, Shanghai, China

**Keywords:** SIRPαFc fusion protein, collagen, macrophage, conjugate, cancer immunotherapy

## Abstract

The SIRPαFc fusion protein can block the immunosuppressive CD47-SIRPα signal between macrophages and tumor cells as a decoy receptor and has demonstrated its immunotherapeutic efficacy in various tumors. However, its clinical application was limited because of the potential hematologic toxicity. The heptapeptide “TKKTLRT” is a collagen-binding domain (CBD) which can bind collagen specifically. Herein, we aim to improve the tumor targeting of SIRPαFc and therefore avoid its unnecessary exposure to normal cells through synthesizing a TKKTLRT–SIRPαFc conjugate. Experiments at molecular and cellular levels indicate that the TKKTLRT–SIRPαFc conjugate-derived collagen-binding affinity and the introduction of CBD did not impact the CD47-binding affinity as well as its phagocytosis-promoting effect on NSCLC cells. *In vivo* distribution experiments showed that CBD–SIRPαFc accumulated in tumor tissue more effectively compared to unmodified SIRPαFc, probably due to the exposed collagen in the tumor vascular endothelium and stroma resulting from the abnormal vessel structure. On an A549 NSCLC nude mouse xenograft model, CBD–SIRPαFc presented more stable and effective antitumor efficacy than SIRPαFc, along with significantly increased CD11b^+^F4/80^+^ macrophages especially MHC II^+^ M1 macrophages within tumors. All of these results revealed that CBD brought a tumor-targeting ability to the SIRPαFc fusion protein, which contributed to the enhanced antitumor immune response. Altogether, the CBD–SIRPαFc conjugate may have the potential to be an effective tumor immunotherapy with improved antitumor efficacy but less non-tumor-targeted side effect.

## Introduction

The incidence and mortality of cancer have increased rapidly in recent years, of which solid tumors account for the majority (1). According to the data from the Global Cancer Observatory, lung cancer accounts for 23.8% of all cancer deaths (2), of which non-small cell lung cancer (NSCLC) accounts for 85% of the diagnoses (3). Studies on antitumor treatments have greatly developed, but an effective therapy is still important and urgent (4). Traditional surgery, chemotherapy, and radiotherapy are initially used to fight against cancer, but the poor survival and high recurrence still remain problems (5). Over the past decade, cancer immunotherapy has grown rapidly. However, cancer cells could always manage themselves to escape from immune surveillance *via* several mechanisms such as upregulation of ligands for immune checkpoints, secreting immunosuppressive cytokines (VEGF, TGF-b1, IL-10, etc.), and epigenetic silencing (4, 6).

T-cell immune checkpoint inhibitors are most common and successful agents used in cancer immunotherapy (6), which would activate the immune system through targeting cancer immune checkpoints such as PD-1/PD-L1, CTLA-4, and block immunosuppression signals (7). Many checkpoint inhibitors (CPIs) such as nivolumab, pembrolizumab, atezolizumab, and ipilimumab have proved their validity in the clinical treatment of NSCLC (8, 9), which mainly target the adaptive immune system and activate T-cell responses (10, 11). However, those CPIs generally only work in the part of patients. Recently, in order to further improve immunotherapy, many studies are focusing on another branch of immune system—innate immune system, which is also crucial to antitumor immunity (12, 13).

Macrophages, known as major immune cells in innate immunity, have also played an important role in cancer immunotherapy (5). The CD47-SIRPα axis is the most thoroughly studied signal pathway regulating phagocytosis by macrophages and other phagocytes, which could be an ideal target (14). CD47 binding to its natural ligand SIRPα (signal regulatory protein alpha) will deliver a “don’t eat me” signal to phagocytes through activating the SHP-1 and SHP-2 signal pathway, inducing phagocytosis inhibition (15). Tumor cells could escape from the innate immunity by expressing high-level CD47 on its surface. Previous studies have shown that blocking the CD47-SIRPα signal could not only restart phagocytosis but also enhance tumor antigen presentation and activate specific antitumor immune response, reacting in both innate and adaptive immunity (16–18).

CD47 is widely expressed in solid tumors (19), promoting multiple agents targeting CD47 or SIRPα, such as anti-CD47 or anti-SIRPα antibodies and fusion proteins (20). SIRPαFc is a fusion protein combining the human SIRPα extracellular domain with the human IgG1 Fc fragment. It could not only effectively block immunosuppressive CD47-SIRPα signals but also induce Fcγ receptor reaction by its Fc fragment. While blocking endogenic CD47-SIRPα signals between tumor cells and phagocytes, SIRPαFc would promote antitumor innate immunity and tumor antigen presenting to activate adaptive immunity (14, 17). Through binding to the Fcγ receptor, SIRPαFc could also mediate antibody-dependent cell-mediated cytotoxicity (ADCC) and antibody-dependent cellular phagocytosis (ADCP) to suppress tumor (5, 20). In previous studies, we have confirmed its antitumor efficacy on non-small cell lung cancer and glioblastoma (21–23).

Erythrocytes and platelets also contain high-level CD47, which help to eliminate senescent cells and maintain the balance (24–27). Thus, agents targeting CD47 may bind to erythrocytes or platelets and the subsequent phagocytosis could induce hematologic toxicity such as hemolytic anemia and thrombocytopenia (28, 29). Although SIRPαFc binds less to CD47 on human erythrocytes than the anti-CD47 antibody, the SIRPαFc-related anemia, thrombocytopenia, and neutropenia still happened in clinically enrolled subjects, thus raising a safety concern (30). Therefore, reducing binding to normal cells including healthy erythrocytes is critical to anti-CD47 immunotherapies.

Collagen is the most common protein in mammals, which appears in almost all the tissues (31), especially the vascular endothelium and tumor stroma. In tumors, vessels were recognized as unhealed wound to be repaired (32), inducing an abnormal and incomplete structure in the vascular wall. Due to the fragmentary vessels, macromolecules in circulation derived from enhanced permeability would keep retention in tumor, which was summarized as EPR (enhanced permeability and retention) effect, while collagen was also exposed around tumor vessels more than in normal tissues (32, 33).

There is a plethora of studies using protein engineering to introduce the collagen-binding domain or protein-binding molecules, which introduces retention or homing of therapeutic proteins in tumors (34, 35) or in other diseases (36, 37). The collagen-binding domain (CBD) is a series of polypeptides which are able to bind collagen. They have a variety of sources such as von Willebrand factor (vWF), fibronectin, or collagenase, containing both natural and artificial sequences (38). “TKKTLRT” is the smallest CBD peptide designed based on the collagenase cleavage site in the collagen-type I α2 chain. This heptapeptide was found to have good tissue penetration and could continuously release a small size of antibody molecules or fusion proteins when binding to the exposed collagen in tumor tissues as conjugates such as CBD–scFv or CBD–Fab fusion protein (39, 40).

Here, we designed and synthesized a TKKTLRT-SIRPαFc conjugate. We propose that the conjugation would confer SIRPαFc collagen affinity. Due to the fragmentary vessels and exposed stroma collagen of solid tumor, our conjugate would specifically accumulate in tumor rather than normal tissues, which represents tumor targeting and leads to better antitumor efficacy by effective and sustainable immune activation and improved safety results from less non-tumor binding (35).

## Materials and Methods

### Synthesis of the CBD–SIRPαFc Conjugate

The construction, expression, and purification of human SIRPαFc fusion protein was performed as previously described (21). Polypeptide “TKKTLRTC” was synthesized by Yuan Peptide (Nanjing) with a purity above 95% detected by HPLC. SIRPαFc was incubated with 15 equivalents of 4-(N-maleimidomethyl)cyclohexane-1-carboxylic acid 3-sulfo-N-hydroxysuccinimide ester sodium salt (Sulfo-SMCC, Sigma-Aldrich, St. Louis, MO, USA) in pH 7.4 PBS for 40 min at room temperature. Dissociative Sulfo-SMCC was removed through 3 × 4 h dialysis in pH 7.4 PBS. The intermediate was then incubated with 10 equivalents of polypeptide “TKKTLRTC” for 1 h at room temperature in the absence of oxygen. A 3 × 4 h dialysis in pure water was performed to eliminate excess polypeptides and desalt. The reaction mixture was frozen at -80°C for 48 h. Freeze-drying was performed to derive CBD–SIRPαFc conjugate powder.

### Identification of CBD–SIRPαFc

Sodium dodecyl sulfate polyacrylamide gel electrophoresis (SDS-PAGE) was first performed to examine whether there was impurity in CBD–SIRPαFc freeze-dried powder. The purified product was dissolved in PBS and then reduced by SDS-PAGE Loading Buffer (Beyotime, P0015, Shanghai, China) according to the manufacturer’s instruction. SDS-PAGE was performed on 12% separating gel. Gel images were acquired with the ChemiDoc™ XRS+ System with Image Lab™ Software (Bio-Rad, Hercules, CA, USA).

Matrix-assisted laser desorption/ionization time-of-flight mass spectrometry (MALDI-TOF-MS) was then performed to determine the exact molecular weight and the ratio of the polypeptide conjugated to SIRPαFc. SIRPαFc and CBD–SIRPαFc were dissolved in pure water and diluted to 1 μg/μl. Proteins were ionized in the matrix of 3,5-dimethoxy-4-hydroxycinnamic acid (sinapic acid, SA), and MALDI-TOF-MS was performed. All spectrograms were collected and analyzed with analysis software Data Explorer™ Software.

### Affinity of CBD–SIRPαFc to CD47 and Collagen

Binding between SIRPαFc/CBD–SIRPαFc and CD47 was detected with Biacore. Sensor S Sensor Chip CM5 (GE Healthcare, Chicago, IL, USA) was activated by EDC-NHS and then caught SIRPαFc/CBD–SIRPαFc proteins on its surface. Human CD47 solution was attenuated into six gradients and flew through the chip in order. A signal–time curve was recorded and analyzed with Biacore T200 (GE Healthcare).

ELISA was performed to measure the affinity between CBD–SIRPαFc and collagen type I. A 96-well ELISA plate was coated with 100 μg/ml collagen type I (Corning, Tewksbury, MA, USA) or 2% BSA at 4°C overnight, then blocked by 2% BSA in PBS-T for 2 h at 37°C. Wells were washed with PBS-T for 5 times and incubated with SIRPαFc or CBD–SIRPαFc in five concentration gradients at 37°C for 2 h. After being washed, wells were incubated with HRP-conjugated anti-human IgG1 Fc fragment antibody at 37°C for 2 h. After a final wash, wells were incubated with TMB substrate at 37°C for 30 min. Absorbance at 450 nm was measured, and an absorbance–concentration curve was drawn in GraphPad Prism 8 software. The *K*_D_ value was calculated through non-linear regression (assuming one-site specific binding).

### Binding of CBD–SIRPαFc to Cancer Cells and Collagen

Flow cytometry was performed to detect the binding of SIRPαFc/CBD–SIRPαFc to cancer cells. 1 × 10^5^ A549 cells were resuspended in PBS (control group), 10 μg/ml SIRPαFc, or CBD–SIRPαFc respectively and incubated at 37°C for 2 h. After being washed with PBS, cells were incubated with DyLight 680-labeled anti-human IgG Fc fragment antibody at 37°C for 1 h. After being washed, cells were resuspended in 200 μl PBS in 96-well plates and detected with Beckman CytoFlex S Flow Cytometer using the APC A700 channel. CytExpert Software was used to analyze the flow cytometry data.

We also performed modified ELISA to test whether CBD–SIRPαFc could bind tumor cells and collagen simultaneously. The 96-well ELISA plate was coated with 100 μg/ml collagen type I (Corning) at 4°C overnight, then blocked with 2% BSA in PBS-T for 2 h at 37°C. Wells were washed with PBS-T for 5 times and incubated with PBS (control group), 100 μg/ml SIRPαFc, or CBD–SIRPαFc at 37°C for 2 h. After washing, 5 × 10^4^ CFDA SE-labeled A549 cells were inoculated and incubated at 37°C for 2 h. After being washed with PBS for 3 times, wells were observed and cells were counted under a laser confocal fluorescence microscope. One-way-ANOVA was performed to analyze the significance in GraphPad Prism 8 software.

### Phagocytosis Test

A phagocytosis test was performed to detect whether CBD conjugation would influence the enhancement of phagocytosis induced by SIRPαFc. 1 × 10^5^ macrophage Ana-1 cells were first inoculated in confocal dishes. After being incubated for 8 to 12 h till the cells had tightly adhered, they were cultivated in serum-free medium for 2 h. After inoculating 2 × 10^5^ CFDA SE-labeled A549, NCI-H1975, or PC-9 cells, cells were respectively incubated in complete medium with no drugs (control group), 10 μg/ml SIRPαFc, or CBD–SIRPαFc in every group for 2 h. Dissociative tumor cells were washed away with PBS, then cells were observed and counted under the laser confocal fluorescence microscope. A phagocytic index (number of phagocytized cancer cells in every 100 ana-1 cells) was calculated to measure the phagocytosis.

### Mice and Cell Lines

Male BALB/c nude mice aged 6 to 8 weeks were obtained from the Shanghai SLAC Laboratory. All procedures involving animals were conducted in accordance with the standards approved by the Animal Ethical Committee of School of Pharmacy at Fudan University. A549 cells, NCI-H1975 cells, PC-9 cells, and Ana-1 cells were kindly provided by Stem Cell Bank, Chinese Academy of Sciences, and cultured according to the instructions in RPMI 1640 medium (BI) containing 10% of fetal bovine serum (Gibco, Grand Island, NY, USA) at 37°C in an incubator with 5% CO_2_.

### Collagen and CBD–SIRPαFc Distribution *In Vivo*

A total of 1 × 10^7^ A549 cells were resuspended in 200 μl PBS then inoculated subcutaneously at the right flank of male BALB/c nude mice aged 6 to 8 weeks. Tumors and paired normal tissues were harvested when reaching 200 mm^3^ and fixed in 4% paraformaldehyde. After embedding in paraffin, tissues were cut into 5-μm-thick sections. Masson-trichrome staining and immunofluorescent staining were performed to label blood vessels and collagen in tumors and normal tissues. In immunofluorescent staining, DAPI (Servicebio, Wuhan, China, G1012) was used to label the cell nucleus. Goat anti-mouse CD31 antibody (Servicebio, GB13063) and Cy3-conjugated donkey anti-goat IgG (H+L) antibody (Servicebio, GB21404) were used to label CD31, which indicated vessels. Rabbit anti-mouse/human collagen I antibody (Servicebio, GB11022-3) and FITC-conjugated donkey anti-rabbit IgG (H+L) antibody (Servicebio, GB22403) were used to label collagen type I.

SIRPαFc and CBD–SIRPαFc were first labeled by FITC and then quantified. Mice bearing tumors reaching 200 mm^3^ were injected with 10 mg/kg FITC-labeled SIRPαFc or CBD–SIRPαFc intraperitoneally. Mice were euthanized with CO_2_ inhalation at 2 or 4 h later after the injection, respectively. Tumors, hearts, livers, spleens, lungs, and kidneys were harvested, and fluorescence signals at 2 h were acquired and measured with Living Image software and counted with GraphPad Prism 8 software. Immunofluorescent staining was performed on tumors and main organs at 4 h to label blood vessels and collagen in tumor tissues. Cell nucleus, CD31, and collagen type I were labeled respectively as described above. Immunofluorescent staining images were acquired with CaseViewer Software.

### *In Vivo* Antitumor Efficacy

An A549 BALB/c nude mouse xenograft model was established as described above. When tumors reached 200 mm^3^ at about 14 days later, mice were injected with 200 μl PBS, SIRPαFc (10 mg/kg), or CBD–SIRPαFc (10 mg/kg) intraperitoneally twice a week. Tumor volume and mouse weight were measured every time before administration. At the 29th day after the first dose, blood was derived from anesthetized mice. Then mice were euthanized with CO_2_ inhalation and tumors, hearts, livers, spleens, lungs, and kidneys were harvested. Part of tumors and organs were fixed in 4% paraformaldehyde then embedded in paraffin for histology and immunology analysis. Tumor volume curves were analyzed with two-way ANOVA in GraphPad Prism 8 software.

### Flow Cytometry

Fresh tumors and spleens were ground and filtered through a 70-μm cell strainer (Falcon, 352350) to prepare single-cell suspension. Antibodies against the following molecules were used according to the manufacturer’s instructions: anti-mouse CD45 (violetFluor 450-labeled, clone: 30-F11, MULTI SCIENCES, Hangzhou, China), anti-human/mouse CD11b (PerCP-Cy5.5-labeled, clone: M1/70, MULTI SCIENCES), anti-mouse F4/80 (PE-Cy7-labeled, clone: BM8.1, MULTI SCIENCES), anti-mouse MHC II (PE-labeled, clone: M5/114.15.2, MULTI SCIENCES), and anti-mouse CD206 (MMR, APC-labeled, clone: C068C2, MULTI SCIENCES). Intracellular staining was performed using FIX & PERM Kit (MULTI SCIENCES) according to the manufacturer’s instructions. Flow cytometry was performed with Beckman CytoFlex S Flow Cytometer. CytExpert Software was used to analyze the flow cytometry data.

### Immunohistochemistry Staining Analysis

Tumors embedded in paraffin were cut into 5-μm-thick sections. Ki67 and F4/80 immunohistochemistry staining was respectively performed to study the proliferation and macrophage infiltration in tumor. Rabbit anti-mouse Ki67 antibody (Servicebio, GB111141) and rabbit anti-mouse F4/80 antibody (Servicebio, GB11027) were used for Ki67 staining and F4/80 staining as primary antibodies, respectively. HRP-conjugated goat anti-rabbit IgG (H+L) antibody (Servicebio, GB23303) was used in both staining as secondary antibody. Images were captured by Inverted Phase Contrast Fluorescence Microscope (Olympus, Tokyo, Japan). Proportions of the positive area were counted by ImageJ software and analyzed by one-way ANOVA in GraphPad Prism 8 software.

### Histology Staining Analysis

Tumors embedded in paraffin were cut into 5-μm-thick sections. Hematoxylin–eosin (H-E) staining was performed to study the necrosis in tumors, which may result from SIRPαFc or CBD–SIRPαFc injection. Images were captured by Inverted Phase Contrast Fluorescence Microscope (Olympus). Focal necrosis in all samples was counted and analyzed by one-way ANOVA in GraphPad Prism 8 software.

### Statistical Analysis

The data were analyzed by GraphPad Prism 8 (GraphPad Software Inc., La Jolla, CA, USA) as described above, and the results were presented as mean ± SD. *p* value < 0.05 was considered statistically significant.

## Results

### CBD Conjugates to SIRPαFc Through Sulfo-SMCC

**Figure 1:**
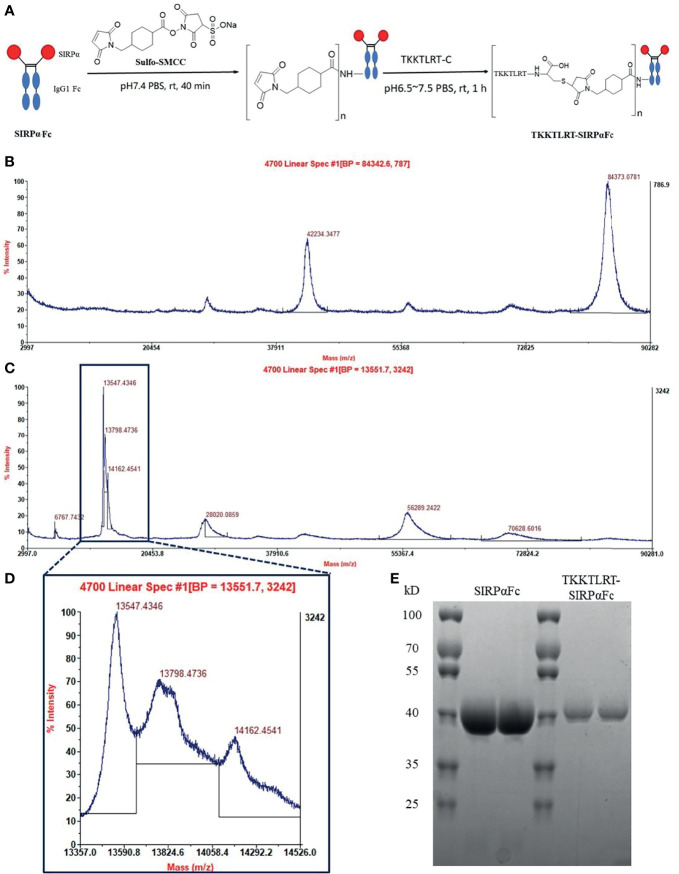
CBD–SIRPαFc conjugate was synthesized and identified. **(A)** Polypeptide “TKKTLRT” conjugates to human SIRPαFc fusion protein with its designed cysteine terminal and a linker Sulfo-SMCC through a two-step reaction. **(B)** The exact molecular weight of SIRPαFc was detected in MALDI-TOF-MS. SIRPαFc weighted 84,373 D and tended to be ionized with two charges, which represented a half mass-to-charge ratio weighting about 42,234 D (data representative of 2 replicates). **(C)** CBD–SIRPαFc was ionized into multiple-charged ions with three (m-z ratio about 28,000), six (m-z ratio about 14,000), or twelve (m-z ratio about 7,000) charges, while some molecules may share charges and represented a non-integral mass-to-charge ratio such as two-thirds (about 56,000) and five-sixths (about 70,000) of the single charge ion (data representative of 2 replicates). **(D)** The main peak (m-z ratio about 14,000) in the CBD–SIRPαFc spectrogram was divided into three sub-peaks. There was a difference of about one-sixth of the total weight of one molecule of Sulfo-SMCC and one molecule of polypeptide “TKKTLRTC” (about 300 D) between each sub-peaks, indicating that one or two molecules of the CBD polypeptide had conjugated to one molecule of SIRPαFc. **(E)** Protein bands were shown in reductive SDS-PAGE (two replicates). SIRPαFc was just below 40 kD. CBD–SIRPαFc showed a simple band at 40 kD above the SIRPαFc band.

CBD polypeptide “TKKTLRT” was conjugated to human SIRPαFc fusion protein through a two-step reaction (**Figure 1A**). In the first step, SIRPαFc linked to the N-succinimide group in Sulfo-SMCC with its dissociative amidogen at room temperature. After dialysis for removing excess linkers, polypeptide “TKKTLRT” with a designed cysteine at the C-terminal was added to the maleimide group of Sulfo-SMCC in the intermediate with its sulfhydryl in the absence of oxygen at room temperature. MALDI-TOF-MS analysis indicated that the CBD conjugation was easier to be ionized than the unmodified protein since its mass-to-charge ratio remained only one-sixth (**Figures 1B, C** **)**. The main peak (m-z ratio about 14,000) in the CBD–SIRPαFc spectrogram was divided into three sub-peaks with a difference of about one-sixth of the total weight of one molecule of Sulfo-SMCC (436 D) and one molecule of polypeptide “TKKTLRTC” (1,452 D; one-sixth of the total weight was about 300 D) between adjacent sub-peaks, indicating that up to two CBD polypeptides were bound to one SIRPαFc protein (**Figure 1D**). SDS-PAGE analysis verified the completeness of CBD–SIRPαFc conjugation that no structural damage happened to SIRPαFc in the reaction, and CBD–SIRPαFc represented a little higher molecular weight than SIRPαFc (**Figure 1E**).

### CBD–SIRPαFc Binds to CD47 and Collagen

**Figure 2:**
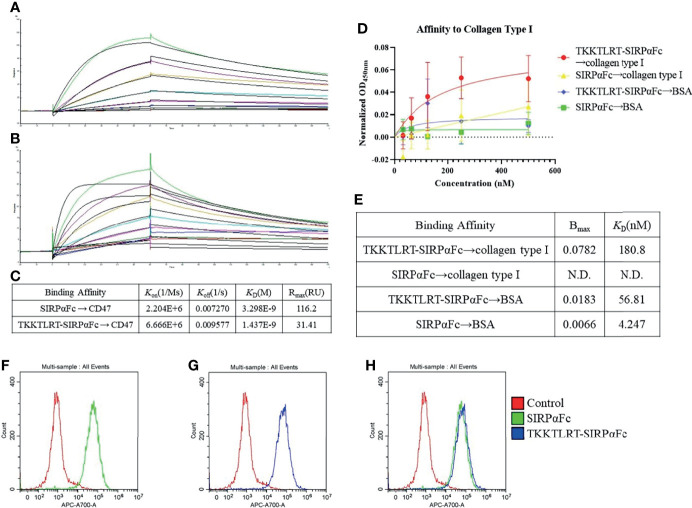
CBD–SIRPαFc showed effective CD47 and collagen affinity at the molecular and cellular levels. Binding of CBD–SIRPαFc to CD47 and to collagen was detected respectively. **(A**–**C)** The affinity of SIRPαFc **(A)** and CBD–SIRPαFc **(B)** to human CD47 was examined, fitted, and analyzed with Biacore. CBD–SIRPαFc represented a similar CD47-binding affinity to unmodified SIRPαFc **(C)**. **(D, E)** A collagen affinity of CBD–SIRPαFc was detected with ELISA. The binding curve was fitted and analyzed through a non-linear curve fit (n = 5, mean ± SD) **(D)**. CBD–SIRPαFc showed a coincident collagen type I affinity as reported, while SIRPαFc did not represent a specific affinity to collagen type I **(E)**. Both SIRPαFc and CBD–SIRPαFc showed limited binding to BSA **(D, E)**. **(F–H)** Binding of SIRPαFc or CBD–SIRPαFc to tumor cells was detected with flow cytometry. Both SIRPαFc **(F)** and CBD–SIRPαFc **(G)** showed significant binding to A549 cells compared to the control group, while their signals were almost at the same level **(H)**.

After the conjugate was successfully synthesized, its impact on the target affinity of SIRPαFc was first characterized. The binding affinity of SIRPαFc and CBD–SIRPαFc to CD47 was detected by Biacore. SIRPαFc and CBD–SIRPαFc were first caught on the activated sensor chip, respectively, then the gradient concentration of human CD47 flew to combine and dissociate (**Figures 2A, B**). CBD–SIRPαFc bound to CD47 with a similar dissociation constant value (*K*_D_ value) to the unmodified protein, which both represented high binding affinities (*K*_D_ = 1.437 × 10^-9^ M for CBD–SIRPαFc and *K*_D_ = 3.298 × 10^-9^ M for SIRPαFc) (**Figure 2C**).

It was reported that CBD peptide “TKKTLRTC” could bind to collagen cross species such as rat, mouse, and human (35, 39, 40). Due to the uncertain molecular weight of collagen and its strong non-specific adsorption to the sensor chip, we performed ELISA to detect the binding of CBD to collagen type I. Five concentrations of SIRPαFc and CBD–SIRPαFc were assayed for collagen binding, and the *K*_D_ value was calculated by non-linear curve fit (**Figure 2D**). The CBD polypeptide conjugated to SIRPαFc showed effective collagen binding ability to collagen type I (*K*_D_ = 180 nM) as previously reported (38), while unconjugated SIRPαFc had no detected binding signals (**Figure 2E**). Both SIRPαFc and CBD–SIRPαFc showed limited binding to BSA as comparison, claiming the specific binding between CBD and collagen type I (**Figures 2D, E** **)**.

### CBD–SIRPαFc Binds to Tumor Cells and Collagen

The abovementioned experiments confirmed the binding affinity of CBD–SIRPαFc to CD47 and collagen at a molecular level. In order to verify the binding at the cellular level, we performed flow cytometry to detect the CD47-binding activity of SIRPαFc and CBD–SIRPαFc on tumor cells. Our previous study proved that A549 NSCLC cells express a high level of CD47 (23). Fluorescence signals revealed that SIRPαFc and CBD–SIRPαFc significantly bound to A549 cells compared to the control group which only incubated with anti-human IgG Fc antibody (**Figures 2F, G** **)**, and they bound to A549 cells with almost the same efficiency (**Figure 2H**).

**Figure 3:**
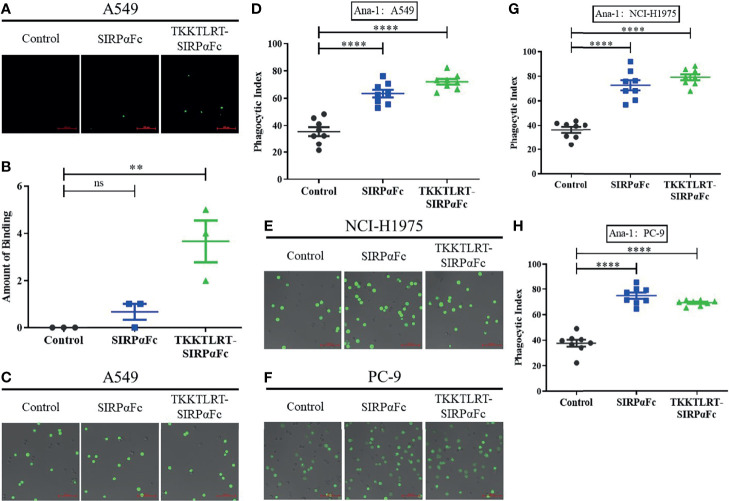
CBD–SIRPαFc retained the coincident *in vitro* effect as SIRPαFc. **(A, B)** CBD conjugation made SIRPαFc derive the ability of binding collagen and tumor cells simultaneously. Significantly more CFDA SE-labeled A549 cells were binding to the CBD–SIRPαFc-incubated group in collagen-coated wells than to the SIRPαFc group (n = 3, mean ± SD ns, no significant, **p < 0.0021). **(C–H)** Phagocytosis of Ana-1 macrophages to A549, NCI-H1975, and PC-9 was detected using the laser confocal fluorescence microscope while tumor cells were labeled with CFDA SE **(C, E, F)**. Macrophages that swallowed tumor cells or not were counted respectively, and the phagocytic index was calculated, in which SIRPαFc and CBD–SIRPαFc showed a similar promoting effect on phagocytosis (n = 8, mean ± SD, ****p < 0.0001) **(D, G, H)**.

Another ELISA was performed to detect SIRPαFc and CBD–SIRPαFc binding to tumor cells and collagen simultaneously. CBD–SIRPαFc first bound to collagen coated on the 96-well ELISA plate, then incubated with CFDA SE-labeled A549 cells and counted under the laser confocal fluorescence microscope (**Figure 3A**). Bindings in wells of each group were counted and analyzed. A549 cells in the CBD–SIRPαFc group were obviously bound and remained in the wells, while no binding was detected in the control group (incubated with no drugs) and very few bindings in the SIRPαFc group, which may be attributed to the adhesion-promoting function of collagen (**Figure 3B**).

All of these results clarified the dual specificity of the designed conjugate, and the introduction of CBD would not influence the affinity of SIRPαFc to its target on tumor cells.

### CBD–SIRPαFc Promotes Phagocytosis *In Vitro*

The function of SIRPαFc in promoting phagocytosis was previously reported, but whether the CBD–SIRPαFc conjugate retained this function still needs to be tested. The effect on macrophage-mediated phagocytosis was detected using the laser confocal fluorescence microscope. We chose three tumor cell lines to test the enhanced phagocytosis induced by CBD–SIRPαFc. After incubation with no drug (control group), SIRPαFc, or CBD–SIRPαFc for 2 h, CFDA SE-labeled A549 cells, NCI-H1975 cells, and PC-9 cells were significantly phagocytosed by Ana-1 macrophages (**Figures 3C, E, F** **)**. The phagocytic index was calculated as described in *Materials and Methods*, in which the SIRPαFc-treated group increased from 35.49 to 63.44 in A549 cells, from 36.39 to 72.71 in NCI-H1975 cells, and from 37.57 to 74.84 in PC-9 cells when compared with the control group. Improved phagocytosis also appeared in the CBD–SIRPαFc group with average phagocytic index values of 72.07, 79.21, and 69.25 in A549, NCI-H1975, and PC-9 cells, respectively, which had no significant difference to the SIRPαFc group (**Figures 3D, G, H** **)**. Therefore, CBD–SIRPαFc retained a similar ability in promoting phagocytosis as SIRPαFc.

### CBD–SIRPαFc Accumulates in Tumor More Quickly Where Collagen Is Abundant

**Figure 4:**
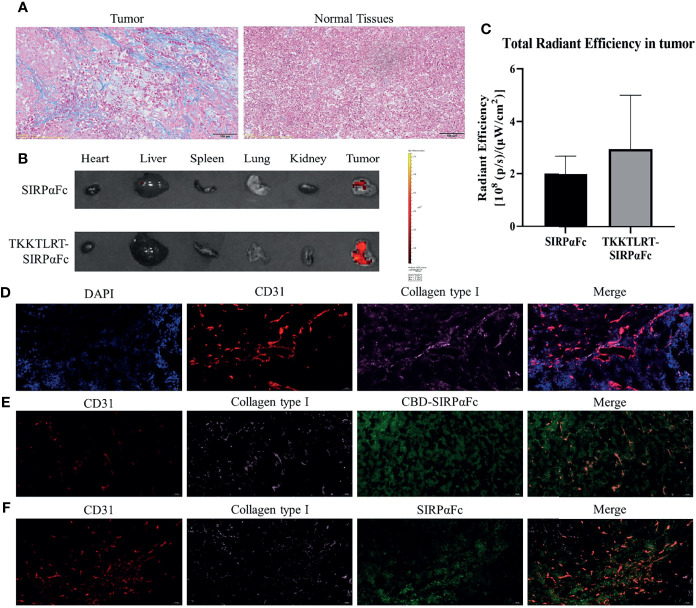
Collagen was abundant in tumors where CBD–SIRPαFc would accumulate. **(A)** Masson-trichrome staining showed collagenous fiber (blue) and muscle fiber (red) in tumors and paired normal tissues. Collagen was largely distributed in tumors compared to normal tissues. **(B, C)** FITC-labeled SIRPαFc and CBD–SIRPαFc distributed in tumor, and main organs at 2 h after intraperitoneal injection were detected, when more CBD–SIRPαFc accumulated in tumor than SIRPαFc (n = 3) **(B)**. Total radiant efficiency in tumor was measured with Living Image software and counted with GraphPad Prism 8 software **(C)**. **(D)** Immunofluorescent staining showed cell nucleus (blue), blood vessels (indicated by CD31, red), and collagen type I (pink) in tumor. Collagen was abundant and tended to be around vessels. **(E, F)** Immunofluorescent staining on tumors at 4 h after injection showed FITC-labeled SIRPαFc **(E)** or CBD–SIRPαFc **(F)** in tumor. Blood vessels indicated by CD31 were stained red and collagen type I was stained pink, while SIRPαFc and CBD–SIRPαFc were green. More CBD–SIRPαFc accumulated in tumor than SIRPαFc and distributed mainly around collagen.

We hypothesized that CBD conjugation would help SIRPαFc to target tumor tissues based on the abundant collagen in tumor stroma. Therefore, we performed Masson-trichrome staining and immunofluorescent staining to determine the content and distribution of collagen in tumor. In both staining experiments, we could find collagen, stained blue in Masson-trichrome staining (**Figure 4A**) and pink in immunofluorescent staining (**Figure 4D**), respectively, which largely existed in tumor tissues compared to paired normal tissues. It is worth noting that in immunofluorescent staining, collagen tended to distribute around the vessels (indicated by CD31 red staining, **Figure 4D**). We then labeled SIRPαFc and CBD–SIRPαFc with FITC and injected it into mice bearing about 200 mm^3^ A549 subcutaneous xenograft. Two hours after administration, CBD–SIRPαFc accumulated more in tumor than SIRPαFc (**Figures 4B, C** **)** did, as reflected by the stronger fluorescent signal observed in CBD–SIRPαFc-treated tumors. No such signal was detected in other main organs. In the immunofluorescent staining of tumor at 4 h after administration, it was noted that more CBD–SIRPαFc could be detected than SIRPαFc in tumor, which mainly distribute around vessels and collagen (**Figures 4E, F** **)**.

### CBD Conjugation to SIRPαFc Improves Antitumor Efficacy *In Vivo*

**Figure 5:**
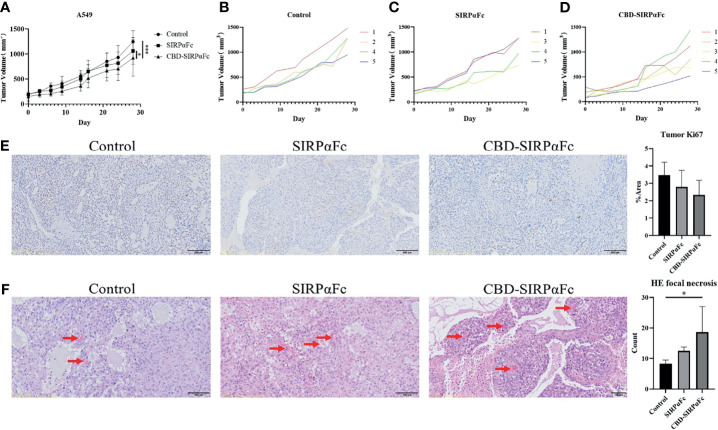
CBD conjugation improved the antitumor efficacy of SIRPαFc *in vivo*. **(A–D)** A total of 1 × 10^7^ A549 cells were resuspended in 200 μl PBS then inoculated subcutaneously at the right flank of each BALB/c nude mouse to establish the xenograft model. Mice were administrated intraperitoneally with PBS, 10 mg/kg SIRPαFc, or CBD–SIRPαFc for 28 days. Tumor volumes were measured and analyzed with two-way ANOVA in GraphPad Prism 8 software between groups (n = 5 for the CBD–SIRPαFc group and n = 4 for the control group and SIRPαFc group, mean ± SD, *p < 0.0332, ***p < 0.0002) **(A)** or in a single group **(B–D)**. One mouse in the control group and one in the SIRPαFc group died before the terminal. **(E)** Ki67 immunohistochemistry staining was performed to study the proliferation in tumor. Tumors in the control group showed a more severe trend of proliferation than the SIRPαFc and CBD–SIRPαFc group. Proportions of the positive area were counted by ImageJ software and analyzed by one-way ANOVA in GraphPad Prism 8 software (n = 5 for the CBD–SIRPαFc group and n = 4 for the control group and SIRPαFc group, mean + SD). **(F)** Hematoxylin–eosin (H-E) staining was performed to study the necrosis in tumors. Focal necrosis indicated by red arrows were more and larger in the SIRPαFc and CBD–SIRPαFc group than in the control group. Focal necrosis in all samples was counted and analyzed by one-way ANOVA in GraphPad Prism 8 software (n = 5 for the CBD–SIRPαFc group and n = 4 for the control group and SIRPαFc group, mean + SD, *p < 0.0332).

In order to examine the *in vivo* antitumor efficacy, we established an A549 subcutaneous xenograft model in nude mice. After administration for 4 weeks, both SIRPαFc and CBD–SIRPαFc exhibited obvious antitumor effects, while CBD–SIRPαFc performed better than SIRPαFc (**Figure 5A**). In a more thorough analysis, suppression on tumor growth varied more widely on each mouse in the SIRPαFc group as compared to the control group (**Figures 5B, C** **)**, while most of the mice in CBD–SIRPαFc derived stable antitumor efficacy (**Figure 5D**). In Ki67 immunohistochemistry staining (**Figure 5E**) and H-E staining (**Figure 5F**), tumors proliferated a little faster in the control group than in the SIRPαFc and CBD–SIRPαFc group (**Figure 5E**), while more focal necrosis appeared in CBD–SIRPαFc and SIRPαFc than in the control group (**Figure 5F**).

### CBD Conjugation to SIRPαFc Enhances Antitumor Immunity

**Figure 6:**
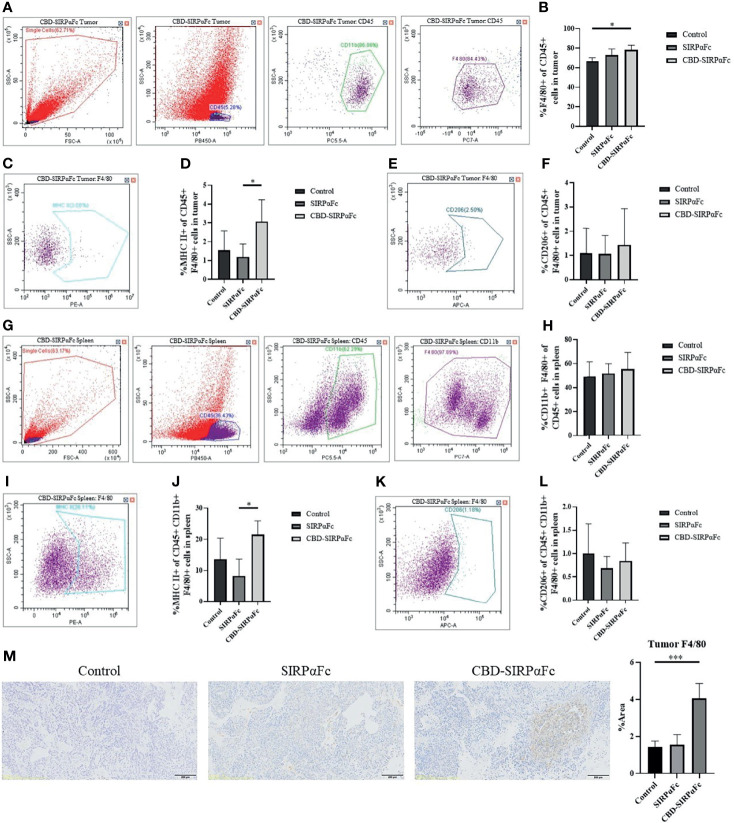
CBD conjugation enhanced antitumor immunity. **(A–L)** Flow cytometry was performed to study the immune cell infiltration in tumors and spleens. **(A–F)** Macrophages in tumors were recognized by CD45^+^CD11b^+^F4/80^+^ signals **(A)**. The percentage of F4/80^+^ cells in total CD45^+^ cells was analyzed, showing more macrophage infiltration in tumor in the CBD–SIRPαFc group than the SIRPαFc and control group **(B)**. M1 and M2 macrophages were also analyzed, in which MHC II^+^ M1 macrophages **(C)** and CD206^+^ M2 macrophages **(E)** were labeled. MHC II+ M1 macrophages were more in the CBD–SIRPαFc group **(D)**, supporting better tumor antigen presentation. M2 macrophages were maintained in all groups **(F)** (n = 5 for the CBD–SIRPαFc group and n = 4 for the control group and SIRPαFc group, mean + SD, *p < 0.3332). **(G**–**L)** Macrophages in spleen were also selected as described above **(G)**, and the percentage of CD11b^+^F4/80^+^ macrophages in total CD45^+^ cells represented the same but weaker trend as in tumor **(H)**. MHC II^+^ M1 macrophages and CD206^+^ M2 macrophages in spleens were labeled as in tumors **(I, K)**. High frequency of MHC II^+^ M1 macrophages appeared in the CBD–SIRPαFc group **(J)** while CD206^+^ M2 macrophages still represented no significant differences **(L)** (n = 5 for the CBD–SIRPαFc group and n = 4 for the control group and SIRPαFc group, mean + SD, *p < 0.3332). **(M)** F4/80 immunohistochemistry staining was performed to help with studying the macrophage infiltration in tumors. F4/80^+^ signals appeared with a higher frequency in tumors of the CBD–SIRPαFc group, which was coincident to the result of flow cytometry. Proportions of positive area were counted by ImageJ software and analyzed by one-way ANOVA in GraphPad Prism 8 software (n = 5 for CBD–SIRPαFc and n = 4 for the control group and SIRPαFc group, mean + SD, ***p < 0.0002).

To determine if the antitumor immunity happened in the *in vivo* study, we performed flow cytometry to investigate macrophage responses in tumors and spleens. Macrophages in tumors were determined by CD45^+^CD11b^+^F4/80^+^ signals (**Figure 6A**), and percentages of F4/80^+^ cells in CD45^+^ cells were calculated and analyzed (**Figure 6B**). CBD–SIRPαFc treatment increased the higher frequency of F4/80^+^ macrophages in total CD45^+^ cells within tumor than SIRPαFc compared to the control group (**Figure 6B**). For further study, M1 and M2 macrophages were indicated by MHC II and CD206 respectively (**Figures 6C, E** **)**. CBD–SIRPαFc significantly increased the frequency of MHC II^+^ M1 macrophages in CD45^+^F4/80^+^ macrophages within the tumor (**Figure 6D**), but the frequency of CD206^+^ M2 macrophages was maintained in all groups (**Figure 6F**). The increase of MHC II^+^ M1 macrophages may indicate better tumor antigen presentation, leading to its effect on adaptive immunity.

Macrophages in spleen were also analyzed (**Figure 6G**). The frequency of CD11b^+^F4/80^+^ macrophages in total CD45^+^ cells within spleen represented a similar but weaker trend as in the tumor (**Figure 6H**), which suggested that CBD–SIRPαFc mainly improved antitumor immunity within the tumor. Analogously, M1 macrophages in spleen indicated by MHC II (**Figure 6I**) increased in the CBD–SIRPαFc group (**Figure 6J**) while M2 macrophages indicated by CD206 (**Figure 6K**) still remained with no significant differences (**Figure 6L**).

We also performed F4/80 immunohistochemistry staining for tumors. The proportion of the F4/80^+^ area was higher in the CBD–SIRPαFc group than in the SIRPαFc group and control group (**Figure 6M**), which revealed the same conclusion.

## Discussion

In recent years, multiple reagents targeting the CD47-SIRPα axis raised and showed a remarkable antitumor efficacy in multiple solid tumors, some of which were already at the phase I study, supporting the essential effect of regulating the CD47-SIRPα signal in cancer immunotherapy (28, 41, 42). Previously, we determined the antitumor efficacy of SIRPαFc fusion protein and found that SIRPαFc elicited potent macrophage-mediated antitumor efficacy in NSCLC *via* blocking endogenous CD47-SIRPα phagocytosis-suppressive signals and inducing antitumor phagocytosis (21). SIRPαFc also represented significant efficacy in refractory NSCLC with resistance caused by anti-angiogenic therapy (23). However, beyond the effective efficacy, adverse reactions induced by blocking CD47 in non-human primates still remained a potential problem (30). Although it is a common barrier in cancer immunotherapy, adverse reactions in anti-CD47 immunotherapy are still worth the attention because anemia or other side effects being induced by antibodies or fusion proteins’ binding to erythrocytes or other hemocytes also means less antibodies accumulating in the tumor (43). Therefore, tumor targeting seems to be an idea killing two birds with one stone, which would improve SIRPαFc in both antitumor efficacy and less adverse reaction.

The collagen-binding domain brought an effective tumor-targeting strategy, which has already been examined in other antitumor antibodies and fusion proteins (35, 39, 40). Collagen is widely distributed in multiple organs and tissues but is especially abundant in the tumor (31, 33). The abnormal and fragmentary blood vessels in the tumor bring the critical chance for CBD to carrier macromolecules to the exposed tumor (32). Compared with some tumor-specific targets such as CD20 and Her2, the common but pivotal elements for tumor targeting of CBD in solid tumors support it to become a general tumor-targeting method crossing different types of tumors (38).

Through MALDI-TOF-MS, we determined the polypeptide–fusion protein ratio showing that one or two molecules of CBD polypeptides bound to one molecule of the SIRPαFc fusion protein. CBD–SIRPαFc retained the same affinity to CD47 as SIRPαFc, which was detected by Biacore, while it also derived collagen type I affinity due to the addition of CBD, which was confirmed by ELISA. In an *in vitro* study, CBD–SIRPαFc would promote phagocytosis of macrophages toward tumor cells, which was satisfactorily not influenced by CBD conjugation. Further, when injected intraperitoneally, CBD–SIRPαFc accumulated in the tumor more quickly compared to SIRPαFc, while almost no conjugate was detected in other main organs. On the A549 NSCLC nude mouse xenograft model, CBD–SIRPαFc represented better antitumor efficacy than SIRPαFc with significantly increased MHC II^+^ M1 macrophages in tumor and spleen tissues, while M2 macrophages were maintained.

The notable increase of MHC II^+^ M1 macrophages revealed a regulating function of CBD–SIRPαFc in macrophage polarization and antigen presentation. M1 macrophages were regarded as proinflammatory phenotype, which could secrete inflammatory cytokines such as TNF-α and IL-1β (44). In antitumor immunity, M1 macrophages not only promote Th1 response to enhance inflammatory reaction but also improve antigen processing and presentation, as well as costimulatory activation of T cells through upregulating related genes (45). Actually, M1 macrophages combine innate immunity and adaptive immunity. Conversely, tumor-associated macrophages (TAM) suppress antitumor immunity through an anti-inflammatory reaction, which have a similar phenotype or consist of M2 macrophages in different theories (46). In a previous study, anti-CD47 was reported to promote M1 macrophage polarization (47). CBD–SIRPαFc increased the proportion of MHC II^+^ M1 macrophages, supporting its function in promoting antigen presentation and following T-cell activation. CBD–SIRPαFc may elicit M1 macrophages through a lasting Fcγ receptor reaction (48), revealing the essential effect of CBD conjugation. Moreover, the proportion of macrophages increased less in spleens than in tumors, revealing that its immunity induction effect focused on tumors, which also suggested that its less non-tumor binding benefited from tumor targeting.

However, as we could see from the *in vivo* experiments, both SIRPαFc and CBD–SIRPαFc did not gain very significant antitumor efficacy in an A549 NSCLC nude mouse xenograft model, which was probably caused by the absence of T cells. As mentioned above, SIRPαFc and other CD47 blocking therapy perform their functions through both innate immunity and adaptive immunity; thus, the lack of T cells would diminish the antitumor efficacy of SIRPαFc to a certain extent (16, 18). Our present study reported a novel CBD–SIRPαFc conjugate, mainly focusing on the synthesis, identification, and affinity examination, with a preliminary *in vivo* efficacy trial. The detailed mechanism of immunological effects such as promoting antigen presentation and T-cell activation and potentially erythrocyte-sparing properties remained to be further investigated. Further *in vivo* antitumor efficacy study should be performed on the human immune system-reconstructed mouse model to involve the T-cell-activating effect of CBD–SIRPαFc. In addition, we are attempting to derive the CBD–SIRPαFc conjugate with a stable CBD–fusion protein ratio through optimizing reaction conditions, as well as designing integrated CBD–SIRPαFc fusion proteins with a stable combination and ratio in order to solve the potential doubts resulted from the multiple-conjugating ratio in the current conjugate.

In conclusion, we make an artificially designed CBD polypeptide “TKKTLRT” conjugate to the SIRPαFc fusion protein through a simple reaction for the first time. We observed that CBD–SIRPαFc gained collagen affinity while its original CD47 affinity and phagocytosis-promoting effect remained the same, which were examined at both molecular and cellular levels. For the *in vivo* study, CBD–SIRPαFc accumulated more and faster in tumor, which also brought an enhanced antitumor innate immunity and efficacy on nude mouse models. There are still mists in conjugate synthesis, but also the predictable effect on adaptive immunity remained to be explored, which is also the way ahead for our study. Taken together, the present study provides a potential strategy to improve the tumor targeting of the phagocytosis checkpoint inhibitor SIRPαFc fusion protein, therefore avoiding unnecessary exposure to normal cells.

## Data Availability Statement

The original contributions presented in the study are included in the article/supplementary material. Further inquiries can be directed to the corresponding author.

## Ethics Statement

The animal study was reviewed and approved by the Animal Ethical Committee of School of Pharmacy at Fudan University.

## Author Contributions

LY, DJ, JL, and ZM contributed to the conception of the study. JL and ZM performed the experiments. TX and KK contributed to the cell experiments. TX and SW contributed to the animal experiments. WT, XZ, and JF contributed to the design and synthesis of SIRPαFc fusion protein. WZ contributed to the design and synthesis of TKKTLRT-SIRPαFc conjugate. JL, ZM, TX, and LY performed the data analysis and wrote the manuscript. XuH, XiH, DP, and HC helped perform the analysis with constructive discussions. All authors contributed to the article and approved the submitted version.

## Funding

This work was supported by the Scientific and Innovative Action Plan of Shanghai (nos. 20S11901600 and 18431902800) and National Natural Science Foundation of China (No. 81572979).

## Conflict of Interest

Author WT is employed by ImmuneOnco Biopharma (Shanghai).

The remaining authors declare that the research was conducted in the absence of any commercial or financial relationships that could be construed as a potential conflict of interest.

## Publisher’s Note

All claims expressed in this article are solely those of the authors and do not necessarily represent those of their affiliated organizations, or those of the publisher, the editors and the reviewers. Any product that may be evaluated in this article, or claim that may be made by its manufacturer, is not guaranteed or endorsed by the publisher.
